# Supplementary material for: Focus on the Dynamics of Neutrophil-to-Lymphocyte Ratio in Cancer Patients Treated with Immune Checkpoint Inhibitors: A Meta-Analysis and Systematic Review
Source: Cancers (Basel). 2022 Oct 27;14(21):5297. doi: 10.3390/cancers14215297 (PMC9658132; doi:10.3390/cancers14215297)
Supplement: Supplementary file 1 [file cancers-14-05297-s001.zip › cancers-1958944-Supplementary.pdf]

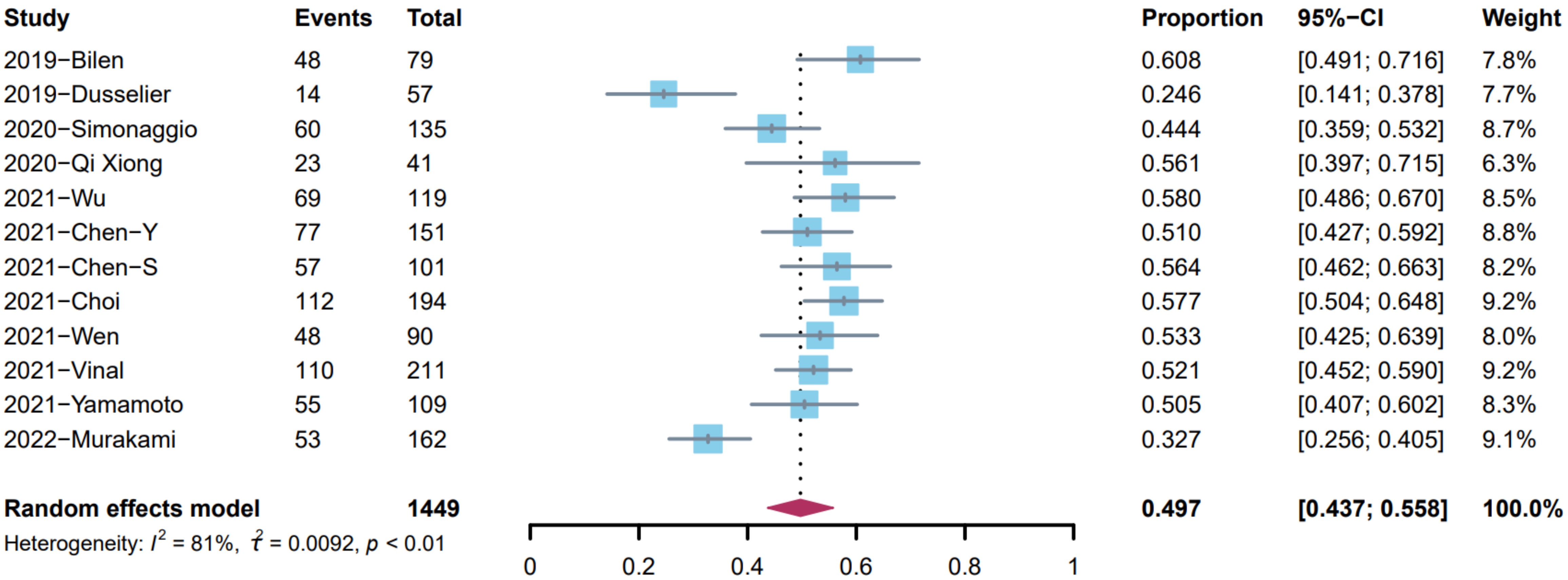

Figure S1. The pooled proportion of patients with increased NLR after immunotherapy.

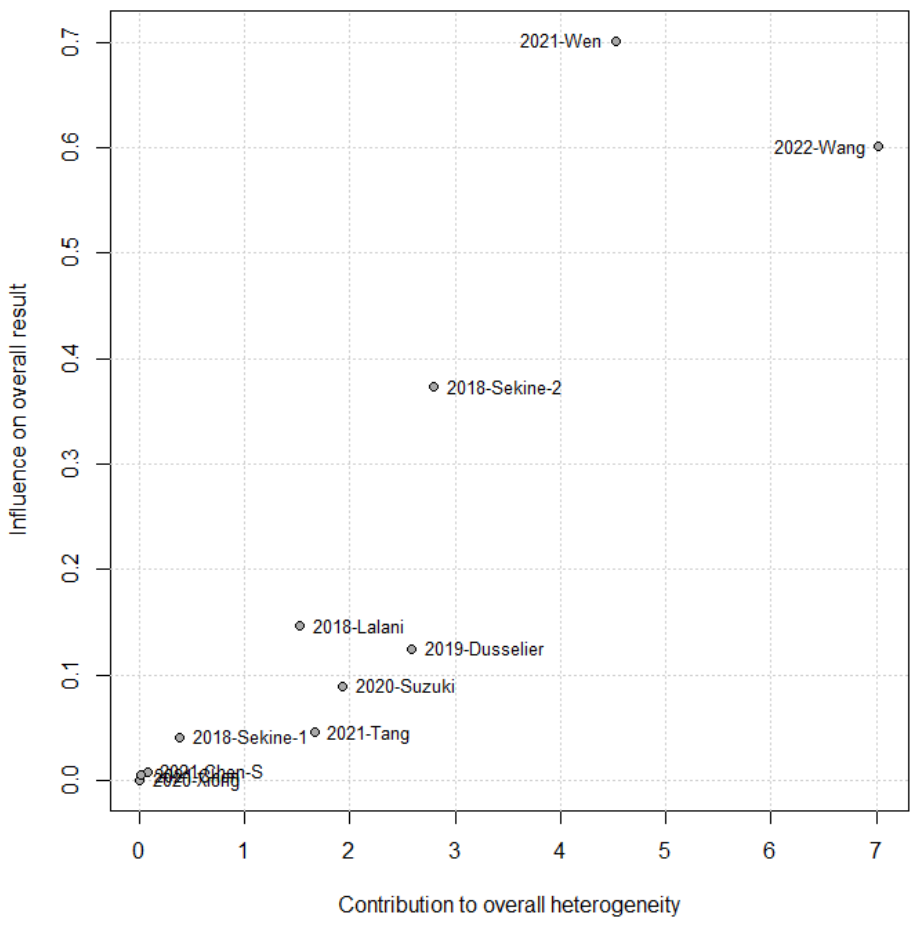

Figure S2. Baujat plot for studies reporting the association between the trends in NLR after ICI treatment and ORR.

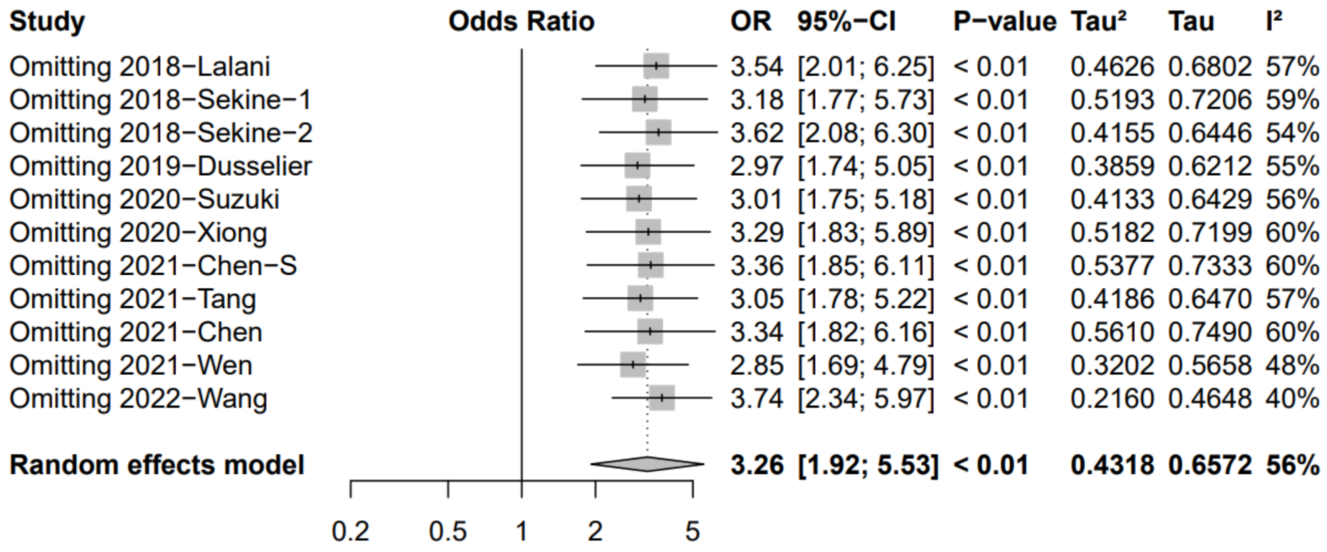

Figure S3. Sensitivity analysis for studies reporting the association between the trends in NLR after ICI treatment and ORR.

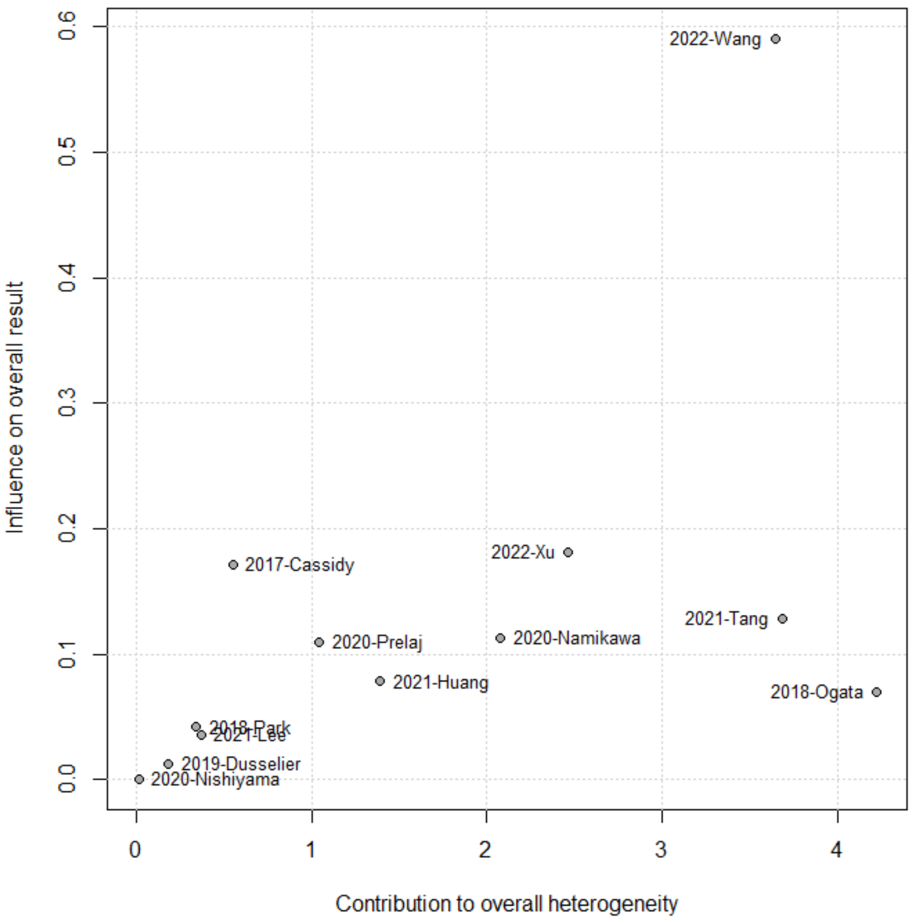

Figure S4. Baujat plot for studies reporting the association between OS and NLR at two different time points with the same cut-off values of NLR.

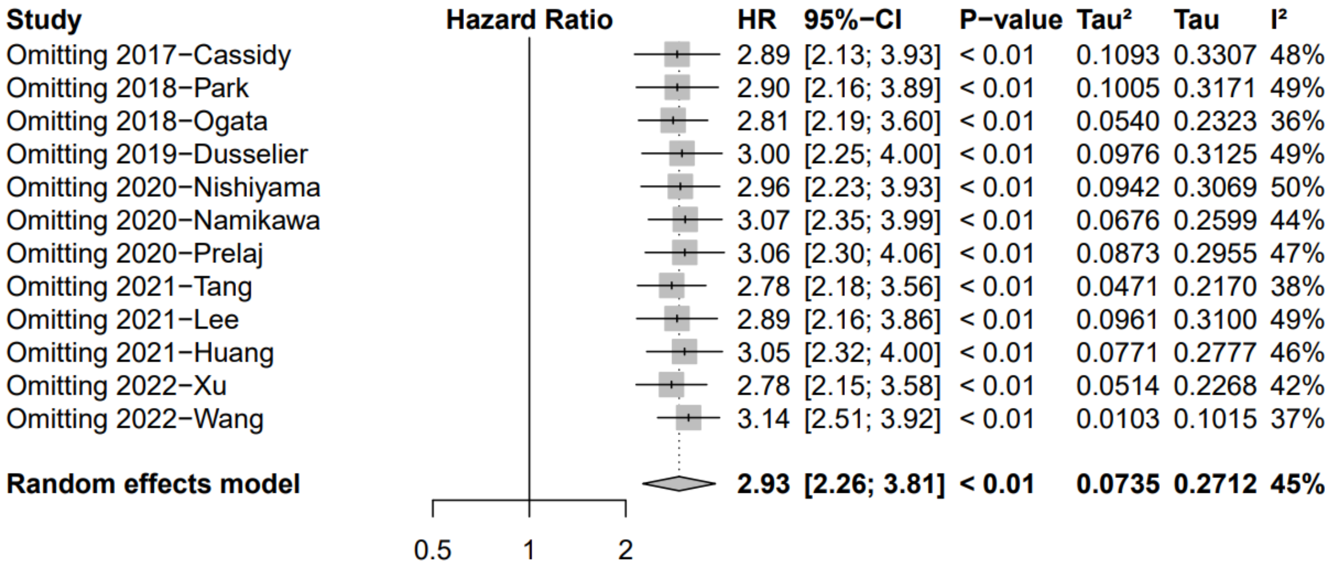

Figure S5. Sensitivity analysis for studies reporting the association between OS and NLR at two different time points with the same cut-off values of NLR.

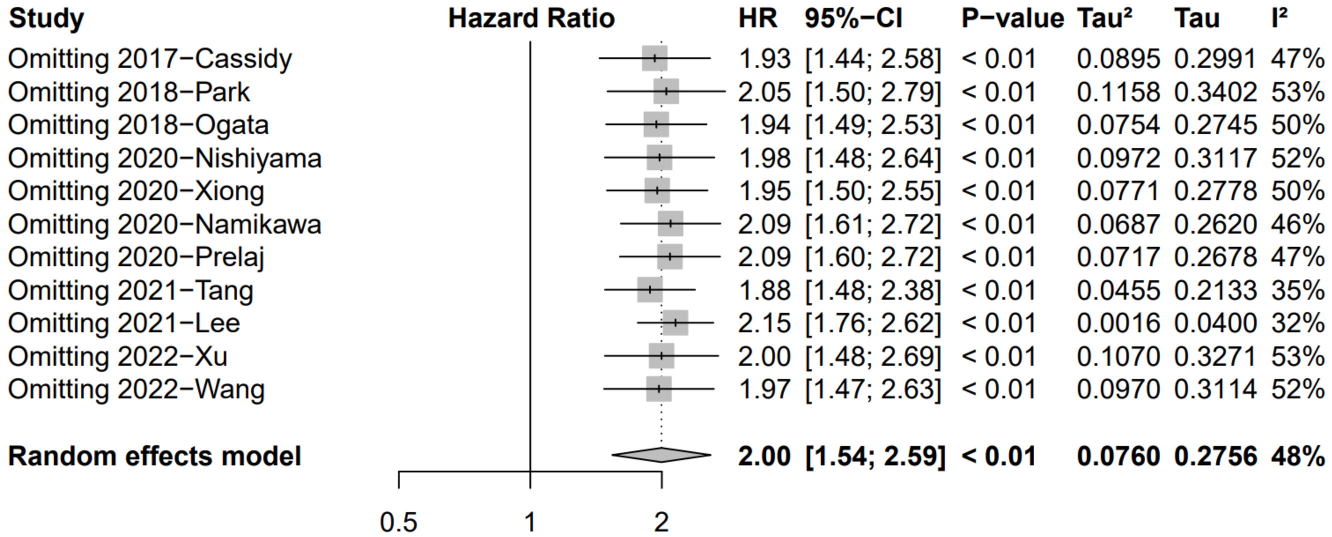

Figure S6. Sensitivity analysis for studies reporting the association between PFS and NLR at two different time points with the same cut-off values of NLR.

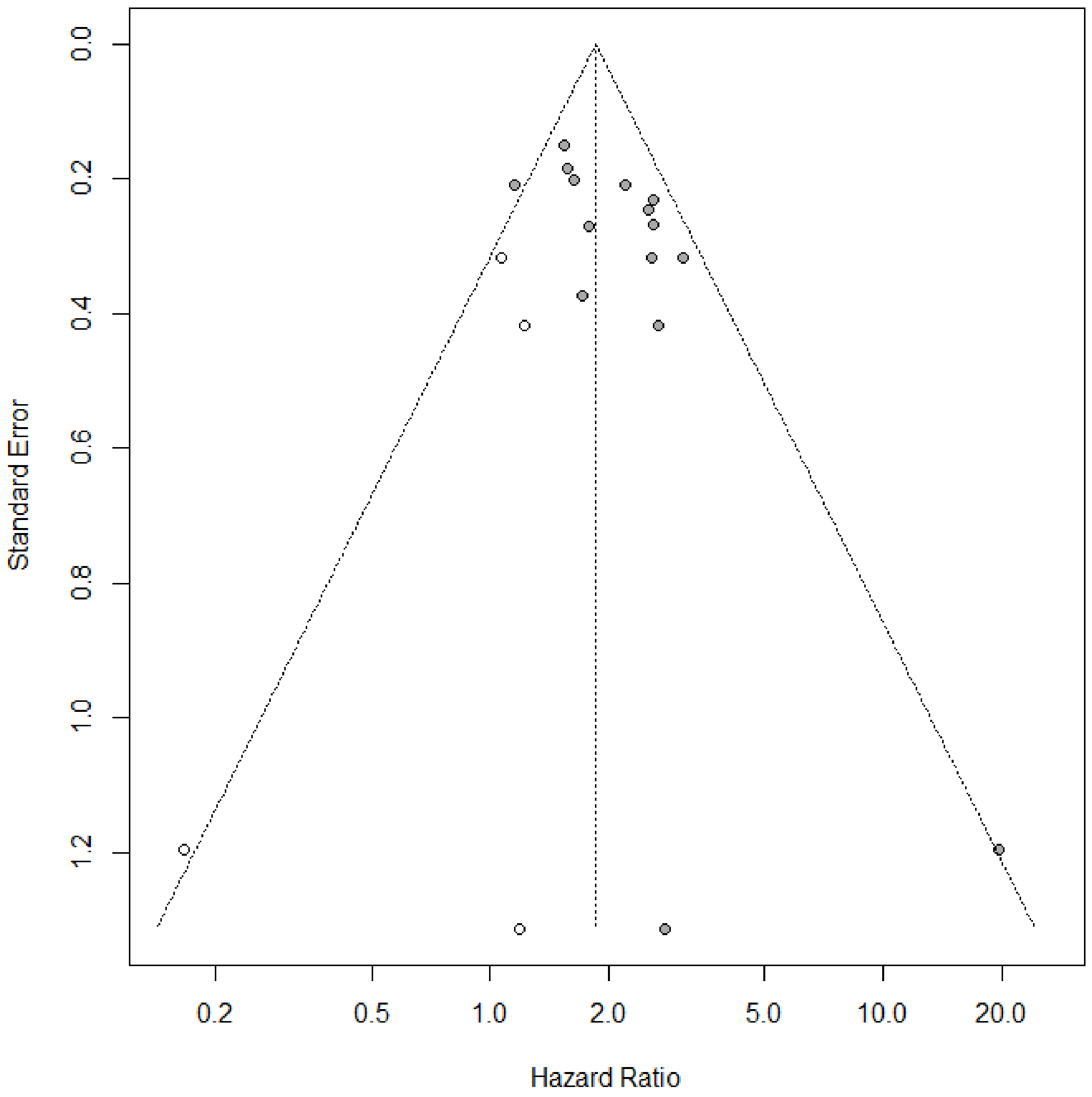

Figure S7. Funnel plot plotted after application of the trim-and-fill method.

Table S1. Studies reporting the association between the changes in NLR and the response to immunotherapy.

<table>
<tr><th>Reference number</th><th>Year</th><th>Author</th><th>Treatment</th><th>Tumor</th><th>The changes of NLR in the patients with response to immunotherapy</th><th>The changes of NLR in the patients without response to immunotherapy</th></tr>
<tr><td>27</td><td>2018</td><td>Alona Zer</td><td>Anti-PD-L1</td><td>NSCLC</td><td>There was a trend toward NLR decrease from baseline to week 8 in patients with response during treatment, and NLR decreased by an average of 1.5 (SD ± 4.2) in patients with objective response.</td><td>There was an increasing trend in NLR by week 8 in patients with stable or progressive disease, and NLR increased by an average of 2.4 (SD ± 6.4) in patients with stable or progressive disease.</td></tr>
<tr><td>37</td><td>2018</td><td>Malaka Ameratunga</td><td>Anti-PD-1 and Anti-PD-L1</td><td>advanced solid tumours</td><td colspan="2">A mixed-effects regression analysis with per-patient random intercept was used to analyse changes in NLR during follow-up. Overall, patients with CR/PR/SD had a lower log transformed NLR (p = 0.03) at study entry (Coef = −0.39; 95% CI = −0.66 to −0.12; p = 0.005 and Coef = −0.39; 95% CI = −0.61 to −0.17; p < 0.001 respectively) compared with patients with progressive disease (PD). There was no evidence for an overall change in transformed NLR during follow-up (Coef = −0.01; 95% CI = −0.08 to 0.05; p = 0.71).</td></tr>
<tr><td rowspan="2">42</td><td rowspan="2">2018</td><td rowspan="2">Monica Khunger</td><td rowspan="2">Nivolumab</td><td rowspan="2">NSCLC</td><td>NLR Before treatment, mean (±SD): 6.1±4.4
NLR after two cycles of nivolumab, mean (±SD): 6.6±11.0</td><td>NLR Before treatment, mean (±SD): 6.5±3.9
NLR after two cycles of nivolumab, mean (±SD): 13.1±22.4</td></tr>
<tr><td colspan="2">There was no significant difference between two groups in the NLR before treatment (6.1 vs 6.5, P=0.66), but the NLR after two cycles of nivolumab in the CR or PR group was significantly lower than that in the SD or PD group (6.6 vs. 13.1, P = 0.09).</td></tr>
</table>

53	2020	Tsutomu Namikawa	Nivolumab	Gastric cancer	NLR Before treatment, median (range): 2.09 (1.59–5.61)<br>NLR 4 weeks after treatment, median (range): 2.19 (2.04–2.62)	NLR Before treatment, median (range):1.83 (0.47–9.42)<br>NLR 4 weeks After treatment, median (range): 2.88 (0.94–12.75)
					There was no significant difference between two groups in the NLR before treatment (2.09 vs 1.83, P=0.59), but the NLR 4 weeks after the treatment in the CR or PR group was significantly lower than that in the SD or PD group (2.19 vs. 2.88, P = 0.044).	
32	2020	Daiki Ikarashi	Nivolumab	mRCC	In the patients with PD, the percent change of NLR at week 6 was significantly higher than patients with SD (P=0.0047) and CR/PR (P=0.0019)	
45	2022	Lin Wang	Camrelizumab	ESCC	NLR Before treatment, median (IQR): 3.14 (2.50-3.74)<br>NLR 8 weeks after treatment, median (IQR): 2.74 (1.98-4.63)<br>In the 16 patients with CR/PR, the NLR level was similar before and after camrelizumab treatment at the first evaluation (3.14 vs 2.74, P=0.718).	NLR Before treatment, median (IQR): 4.27 (2.63-6.57)<br>NLR 8 weeks after treatment, median (IQR): 4.64 (2.92-6.34)<br>In the 32 patients with PD, the median NLR significantly increased from 4.3 (IQR 2.6–6.6) at baseline to 4.6 (IQR 2.9–6.3) (P=0.039) at the first evaluation.

Note. NSCLC: non-small cell lung cancer; ESCC: esophageal squamous cell carcinoma; mRCC: metastatic renal cell carcinoma; NLR: neutrophil-to-lymphocyte; OS: overall survival; PFS: progression-free survival; PD-1: programmed death-1; PD-L1: programmed death ligand-1; IQR: interquartile range; CR: complete response; PR: partial response; SD: stable disease; PD: progressive disease.

Table S2. Studies reporting the association between trends in NLR and prognosis.

Reference number	Year	Author	Treatment	Tumor	Time point	Trend and degree	Outcome
21	2018	Aly-Khan A Lalani	Anti-PD1 and anti-PDL1	mRCC	Baseline and 6 weeks	increase>25%; decrease>25%	OS, PFS and ORR
29	2018	Wungki Park	Nivolumab	NSCLC	Baseline and before the second dose of nivolumab	increase; decrease	OS and PFS
22	2018	Katsutoshi Sekine	Nivolumab	NSCLC	Baseline and 4 weeks	decrease>10%	OS, PFS and ORR
44	2019	Mehmet A Bilen	/	Melanoma, gastrointestinal cancer, Lung/head and neck cancer, Breast cancer and other.	Baseline and 6 weeks	increase; decrease	OS and PFS
39	2019	Matthieu Dusselier	Nivolumab	NSCLC	Baseline and the fourth nivolumab infusions	increase; decrease	ORR
34	2019	Francesco Passiglia	Nivolumab	NSCLC	Baseline and 6 weeks	increase>10%	OS
54	2019	A Simonaggio	Nivolumab	mNSCLC and mRCC	Baseline and 6 weeks	increase; decrease	OS and PFS
28	2020	Kotaro Suzuki	Nivolumab	mRCC	Baseline and 4 weeks	decrease>25%	PFS and ORR

46	2020	Daichi Tamura	Pembrolizumab	Urothelial carcinoma	Baseline and 6 weeks	increase>6.12%	PFS
26	2020	Takuto Shimizu	Pembrolizumab	Metastatic urothelial carcinoma	Baseline and 4 weeks	increase>15%	OS and PFS
23	2020	Yumiko Ota	Nivolumab	Gastric cancer	Baseline, 4 weeks (PFS) and 8 weeks (OS)	increase>2 units	OS and PFS
47	2021	Shixue Chen	Nivolumab, pembrolizumab and others	NSCLC	Baseline and 6 weeks	increase; decrease	OS, PFS and ORR
20	2021	Yuzhong Chen	Pembrolizumab, Sintilimab and Toripalimab	NSCLC	Baseline and 6 weeks	increase; decrease	PFS and ORR
18	2021	Jeong Uk Lim	Nivolumab, pembrolizumab, and atezolizumab	NSCLC	Baseline and 2 weeks	increase>1 unit	PFS
43	2021	Qi Xiong	Nivolumab, pembrolizumab, atezolizumab, and toripalimab	SCLC	Baseline and 6 weeks	increase; decrease	ORR
30	2021	Yin Tang	Anti-PD1 and anti-PDL1	NSCLC	Baseline and 6 weeks	increase>20%; decrease>20%	OS, PFS and ORR
50	2021	Jwa Hoon Kim	Nivolumab and pembrolizumab	Esophageal Squamous Cell Carcinoma	Baseline and after a treatment cycle	increase>40%	OS and PFS

33	2021	Won-Mook Choi	Nivolumab	HCC	Baseline and 4 weeks	increase; decrease	OS and PFS
40	2021	Pei Yi Lee	Nivolumab, pebrolizumab, atezolimumab, avelumab, durvalumab, and tremelimumab	Lung cancer, colorectal cancer, nasopharyngeal carcinoma, gastric cancer, hepatocellular carcinoma.	Baseline and 6 weeks	increase; decrease	OS and PFS
41	2021	Xianbin Wu	Anti-PD1	Esophageal squamous cell carcinoma	Baseline and 6 weeks	increase	ORR
48	2021	D Viñal	/	Lung cancer, melanoma, kidney cancer, bladder cancer, others	Baseline and before a treatment cycle	increase; decrease	OS and PFS
52	2021	Jin Shang	Nivolumab, pembrolizumab, atezolimab, ipilimumab, and sintilimab.	Pancreatic cancer	Baseline and after 2 doses	decrease>10%	OS and PFS
51	2022	Yusuke Murakami	Nivolumab	NSCLC	Baseline and 4 weeks	increase; decrease	OS
45	2022	Lin Wang	Camrelizumab	Esophageal squamous cell carcinoma	Baseline and 8 weeks	decrease>20%	OS, PFS and ORR

8	2022	Deniz Can Guven	Nivolumab, atezolizumab, pembrolizumab, ipilimumab and avelumab	RCC, melanoma, NSCLC and other.	Baseline and 4 weeks	increase>10%	OS and PFS
19	2022	Shaodi Wen	Pembrolizumab, sintilimab, toripalimab	NSCLC	Baseline and 6 weeks	increase; decrease	ORR

Note. NLR: neutrophil-to-lymphocyte ratio; NSCLC: non-small cell lung cancer; SCLC: small cell lung cancer; mNSCLC: metastatic non-small cell lung cancer; RCC: renal cell carcinoma; mRCC: metastatic renal cell carcinoma; HCC: hepatocellular carcinoma; OS: overall survival; PFS: progression-free survival; ORR: objective response rate; PD-1: programmed death-1; PD-L1: programmed death ligand-1

Table S3. Studies that gave the values of baseline and post-treatment NLR with the same cut-off values.

Reference number	Year	Author	Treatment	Tumor	Cut-off values	Time point	Outcome
35	2017	Michael R Cassidy	Ipilimumab	Melanoma	5	Baseline and 6 weeks	OS and PFS
29	2018	Wungki Park	Nivolumab	NSCLC	5	Baseline and before the second infusion	OS and PFS
38	2018	Takatsugu Ogata	Nivolumab	Gastric cancer	5	Baseline and after the first infusion	OS and PFS
39	2019	Matthieu Dusselier	Nivolumab	NSCLC	5	Baseline and after the fourth infusion	OS
36	2020	Naotaka Nishiyama	Nivolumab	mRCC	3	Baseline and 4 weeks	OS and PFS
53	2020	Tsutomu Namikawa	Nivolumab	Gastric cancer	2.5	Baseline and 6 weeks	OS and PFS
31	2020	Arsela Prelaj	Nivolumab and pembrolizumab	NSCLC	4	Baseline and the third treatment cycle	OS and PFS
43	2021	Qi Xiong	Nivolumab, pembrolizumab, atezolizumab, and toripalimab	SCLC	5	Baseline and 6 weeks	PFS

25	2020	Lan Huang	Nivolumab, pembrolizumab, atezolizumab and ipilimumab	NSCLC	5	Baseline and the fourth treatment cycle	OS
30	2021	Yin Tang	Anti-PD1 and anti-PDL1	NSCLC	5	Baseline and 6 weeks	OS and PFS
40	2021	Pei Yi Lee	Nivolumab, pebrolizumab, atezolimumab, avelumab, durvalumab, and tremelimumab	Lung cancer, colorectal cancer, nasopharyngeal carcinoma, gastric cancer, hepatocellular carcinoma.	5	Baseline and 6 weeks	OS and PFS
24	2022	Jianming Xu	Sintilimab	Esophageal squamous cell carcinoma	3	Baseline and 6 weeks	OS and PFS
45	2022	Lin Wang	Camrelizumab	Esophageal squamous cell carcinoma	4	Baseline and 8 weeks	OS and PFS

Note. NLR: neutrophil-to-lymphocyte ratio; NSCLC: non-small cell lung cancer; SCLC: small cell lung cancer; mRCC: metastatic renal cell carcinoma; NLR: neutrophil-to-lymphocyte; OS: overall survival; PFS: progression-free survival; PD-1: programmed death-1; PD-L1: programmed death ligand-1
